# Older Adults Mental Health (OAMH) Services Across Wales – A National Service Evaluation Survey

**DOI:** 10.1192/bjo.2024.512

**Published:** 2024-08-01

**Authors:** Amr Romeh, Patrick Chance, Amrita Varanasi

**Affiliations:** Aneurin Bevan University Health Board, Newport, United Kingdom

## Abstract

**Aims:**

Theoretically, OAMH services would be similar across all Welsh health boards but the reality can differ. To my knowledge, such data about OAMH services across Wales does not exist in a structured way. So I aim to explore these similarities/differences across different Welsh health boards.

**Methods:**

A 20-question google forms survey was sent to 65 doctors from the seven Welsh health boards including long-term trust-grade doctors, middle-grade/SpRs and consultant psychiatrists. It is a box-ticking survey with comment fields for sharing potential thoughts.

**Results:**

Response rate is 50.7% (33/65) with representation from all health boards. Consultants represent 72.2% of responses.

There is some variation in MDT members. Large variation shows in number of organic and functional beds. 33.3% have wards with mixed-type patients. 66.7% have separate wards for each cohort of patients. 30.3% have no inpatient duty but those who have (69.7%), show a varied number of inpatients. Only one sector has long-stay beds.

63.6% indicate that outpatient duty is divided into functional and memory services. Number of clinics differs hence varied numbers of patients.

57.6% have support of COTE on request, some have their regular attendance and some struggle to have their support. 66.7% indicate that care-coordinators are CPNs, otherwise they are OTs, social workers, psychologists or consultants.

75.8% find it better to have one team providing care for the same patient in the community and as inpatients; one major factor being continuity of care.

72.7% have medical students shadowing them in a structured way.

63.6% do not have specialized clinics in the community, others state they have clinics for lithium, clozapine, depot, S117 after-care, antipsychotic review, MCI or neuropsychiatry.

60.6% of liaison services are old-age specific. Some comments state that even in ageless services, they have an older adult psychiatry consultant. One comment states that there are designated nurses to each age group but the consultant is not “old-age trained”.

90.9% of memory services are run by psychiatric service; 9.1% by other departments.

**Conclusion:**

Variations are not only across different health boards but also in-between sectors in each health board. Responses indicate variation in structure of inpatient, outpatient, liaison service and community specialized clinics. There are different levels of support from COTE. Structured medical students’ placements are shown in majority of responses. Finally, satisfaction of subconsultant-level doctors is clear by their wish to continue in the same field.

